# Comparison between premortem histopathology findings in rats with and without traumatic brain injury: prospective application in forensic medicine

**DOI:** 10.12688/f1000research.140718.2

**Published:** 2024-05-22

**Authors:** Taufik Suryadi, Kulsum Kulsum

**Affiliations:** 1 Department of Forensic Medicine and Medicolegal, Dr. Zainoel Abidin Hospital, Banda Aceh, Aceh, 23126, Indonesia; 2 Department of Forensic Medicine and Medicolegal, Faculty of Medicine, Syiah Kuala University, Banda Aceh, Aceh, 23111, Indonesia; 3 Department of Anesthesiology and Intensive Therapy, Dr. Zainoel Abidin Hospital, Banda Aceh, Aceh, 23126, Indonesia; 4 Department of Anesthesiology and Intensive Therapy, Faculty of Medicine, Syiah Kuala University, Banda Aceh, Aceh, 23111, Indonesia

**Keywords:** forensic medicine, histopathology findings, premortem, TBI

## Abstract

**Background:**

The aim of this study was to compare pre-mortem histopathology findings in rats with and without traumatic brain injury (TBI) and its prospective application in forensic medicine.

**Methods:**

This study involved 12 rats with 6 rats for each treatment group. This type of study is a laboratory experimental study with two independent groups design. The first group were rats that did not experience TBI. The second group was a group of rats with TBI. The subjects of this study were Rattus norvegicus rats, adult males, 4-8 weeks old, weighing 150-200 grams. On the 8 ^th^ day after the rats experienced traumatic brain injury, the rats were then euthanized using the cervical dislocation method, after euthanasia the rats were given craniotomy and brain tissue was taken for histopathology examination.

**Results:**

The description of histopathology changes in the brain organs in the group of rat without TBI found that neuron cells looked normal although there were also degeneration (21.16 ± 2.56/FV), necrosis (5.75 ± 0.98/FV), apoptosis (2.91 ± 0.80/FV), congestion ( 0.91 ± 0.49/FV), inflammatory cells (4.58 ± 1.15/FV) and hemorrhage (2.41 ± 1.11/FV). Changes in the rat traumatic brain injury group showed a lot of damage to neuron cells in the form of degeneration (48.41 ± 3.27/FV), necrosis (36.66 ± 2.89/FV), apoptosis (18.91 ± 1.24/FV), congestion (2.50 ±0.31/FV), inflammatory cells (11.41 ± 1.71/FV) and hemorrhage (10.08 ± 2.17/FV). Based on the results of statistical analysis, it can be seen that in all parameters there is a significant difference (p ≤ 0.001).

**Conclusions:**

The premortem histopathology findings in rats with and without TBI which can be used for the benefit of forensic medicine in determining whether TBI is present or not. It is necessary to look more closely at the histopathology changes in the form of necrosis, apoptosis and hemorrhage.

## Introduction

Determining the presence of trauma in forensic medicine is very important in assisting the criminal investigation process. ^1^ Trauma cannot always be determined through external examination, so an internal examination (autopsy) is needed. ^2^ Macroscopic examination of organs or body tissues that have been traumatized sometimes does not show satisfactory results. It is necessary to carry out a microscopic examination so that evidence of trauma is more accurate. ^3^ Forensic pathologists are always challenged to prove whether a person died as a result of trauma or not, and the opinion of the forensic pathologists is very important for medico legal purposes. ^1^^,^^4^ In determining the cause of death, including traumatic brain injury (TBI) is usually carried out by internal examination (autopsy), but this is still incomplete because it only leads to the level of epidemiological causes of death not yet leading to causality. ^3^ Histopathology changes in injuries such as TBI are still being discussed. In general, if TBI occurs when a victim has not died (premortem), then the histopathology changes will be obvious, but if there is postmortem, then there is no inflammatory response. ^4^

Although research on the human body has often been carried out in forensic medicine, the accuracy of whether or not trauma is present still needs to be improved. Given the similarities in anatomy and physiology with humans, experimental animals can be considered in translation research from biomedical to clinical. The frequency of using experimental animals in forensic medical research published in scientific journals is around 4%. ^5^ In order to investigate a premortem histopathology changes in TBI, rats were euthanized. The definition of a premortem is a condition that occurs shortly after death, but the time limit is still controversial. ^6^

TBI induces a complex cascade of immunologic or tissue inflammatory responses that mimic ischemic reperfusion injury. ^7^ Primary head injury and secondary head injury activate the release of cellular mediators in the form of prostaglandins, pro inflammatory cytokines, and free radicals. In secondary injuries, there are disturbances in the processes of metabolism and homeostasis of brain cell ions, intracranial hemodynamics, and cerebro-spinal fluid (CSF) compartments which begin after the trauma but are not clinically apparent immediately after the trauma. ^7^^,^^8^

Deaths due to TBI are some of the most common cases encountered by forensic pathologists. TBI is routinely implicated in cases classified as accidents, as well as in suicides and homicides. Recently, cases of traumatic brain injury have increased in both developed and developing countries. Therefore, it is very useful for evaluating the clinical and pathological features of head injuries. To carry out an investigation of the causes of death in traumatic brain injury cases, a comprehensive examination is needed, one of which is histopathology findings. Histopathology changes in deaths from traumatic brain injury are very important to determine the qualification of whether there was a TBI before death. Therefore, it is necessary to carry out research related to the comparison of premortem histopathology findings using experimental rats with and without traumatic brain injury.

The aim of this study was to compare the results of premortem histopathological findings in animal model with or without traumatic brain injury. The target of the research is for forensic pathologists to be better able to provide evidence of traumatic brain injury to investigators so that the investigation process is optimally successful. The significance of this research is that it provides additional information for forensic pathologists in determining the mechanism of death if this research can later be applied to humans.

## Methods

### Study design

Researchers conducted laboratory experiments on animals with two independent groups design by comparing the first group with the second group. The first group were rats that did not have traumatic brain injury administered to them. The second group is a group of rats that have been administered of traumatic brain injury.

### Animal model

The subjects of this study were 12 healthy Rattus norvegicus rats (6 each group), male, adult, 4-8 weeks old, weighing 150-200 grams, no visible anatomical abnormalities, no signs of previous infection, no other diseases. Exclusion criteria: deformed male white rat (Rattus norvegicus) Wistar strain, rats during the study did not want to eat. Prior to treatment, the fur was given a number to be used in a simple randomization process, then adaptation was carried out for 10 days, homogenization was carried out in the cage, the temperature in the cage was adjusted to room temperature, and every day the rats were fed BR 1 feed. BR 1 is a standard feed for animal model that contains a number of nutrients, such as crude protein, fat and crude fiber.

### Administration of traumatic brain injury

**Figure 1.**
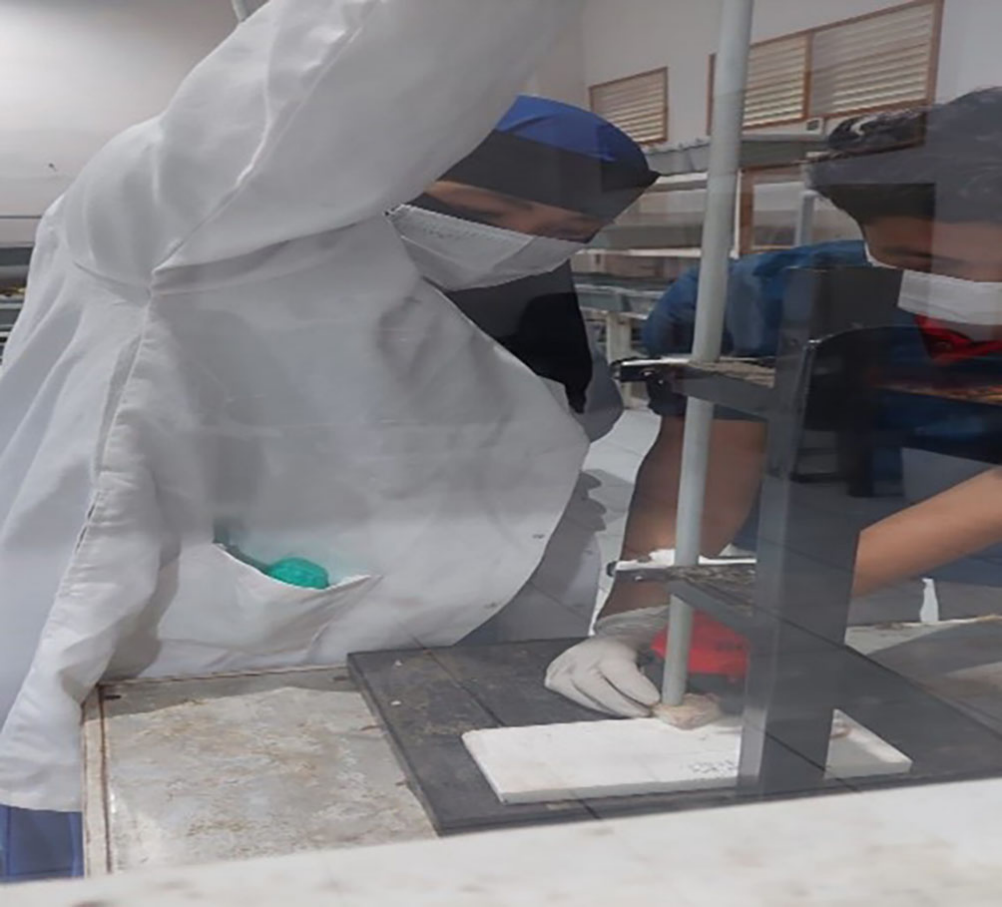
Documentation when the Feeney weight drop model was carried out.

The rat model of traumatic brain injury was made using the Feeney weight drop mode method ( Figure 1) by shaving the scalp of the male rat Rattus norvegicus and then topical anesthesia was given before incision. The incision was done on the right frontoparietal measuring 4 mm to get bone exposure. The center of the skin opening was 1.5 mm posterior to the bregma and 2.5 mm lateral to the midline, then the rat was dropped from a height of 1 meter with a 2.5 mm diameter load. The administration of TBI in the study is a routine procedure for animal cases within the animal laboratory of Veterinary Faculty of Syiah Kuala University that implemented the principles of replacement, reduction, and refinement (3R) and also the principles of five freedom from Farm animal welfare council UK, 1993 which consist of (1) freedom from hunger and thirst, (2) freedom from discomfort, (3) freedom from pain, injury and disease, (4) freedom from fear and distress, and (5) freedom to express natural behavior. The procedures are in accordance with the 2017 Health Research Ethics Committee Guideline by the Ministry of Health of Republic of Indonesia and the 2016 Council for International Organization of Medical Sciences (CIOMS) ethical guidelines.

### Surgical procedure

On the 8 ^th^ day after the rats experienced traumatic brain injury or not, the rats were then euthanized using the cervical dislocation method, after euthanasia the rats were given craniotomy and the brain tissue was taken. The brains taken were weighed and recorded. The entire procedure is performed by the same operator using micro surgical instruments.

### Histopatology preparation

After weighing, the part of the brain case group and control group are included in a 10% neutral buffered formalin (NBF) fixation solution with a ratio of 1:9 for making premortem histopathology preparations. The fixation solution used was 10% formalin (a mixture of 10 cc of 40% formaldehyde with 90 cc of distilled water). The brain organ was immersed in a 10% neutral buffer formalin solution, which serves to provide a hard consistency so that the tissue can be sliced thinly and to provide staining and optical differentiation. The tissue was fixed for 24 hours. Continued with cutting (trimming) organ sample into smaller size. Dehydration using graded ethanol starting from 70%, 90%, and 96%. Purification with xylol solvent. The cleaning process was done by immersing the tissue in a solution of xylene I and II for two hours. Paraffin infiltration is soaking the tissue in melted paraffin at a temperature of 58-60°C for 30 minutes to 6 hours, to remove the clearing agent from the tissue so as to make the tissue resistant to cutting. Blocking is the process of freezing a preparation so that it can be cut with a microtome. Sectioning is cutting the block of preparations using a microtome. The paraffin block was then cut using a rotary microtome with a thickness of 5 μm. Finally hematoxylin eosin (HE) staining and observed under a microscope with 400× magnification. Step by step description of histopathology preparation can be found on Protocols.io).

### Statistical analysis

The research results were analysed using the Shapiro-Wilk normality test. Statistical analysis was carried out using ANOVA (analysis of variance) on normally distributed data. For data that is not normally distributed, the Kruskal Wallis hypothesis test is carried out. Post hoc test analysis was carried out on different data pairs. This research is significant if the p value < 0.05. All the statistical analyses were carried out using SPSS (IBM, New York, US). A proprietary free alternative that can be suggested is PSPP.

### Ethics approval

This study was approved by the Health research ethics committee at the Faculty of Medicine, Universitas Syiah Kuala No. 038/EA/FK/2023.

## Results

This study involved 12 rats for each treatment group with and without traumatic brain injury. Table 1 shows the development of rat body weight before acclimatization, after acclimatization and rat brain weight after treatment. Macroscopically, there were differences between the two treatment groups as can be seen in Figure 3. The histopathology findings in both groups of rats can be seen in Figure 4. Quantitatively, the processes of degeneration, necrosis, apoptosis, congestion, inflammatory cells and hemorrhage in the two groups can be seen in Table 2. The average quantitative processes of degeneration, necrosis, apoptosis, congestion, inflammatory cells and hemorrhage in all groups can be seen in Table 3.

**Table 1.** Development of rat body weight before acclimatization, after acclimatization and rat brain weight after treatment.

No	Rat body weight (grams) before acclimatization		Rat body weight (grams) after acclimatization		Rat brain weight after treatment (grams)	
	Not TBI	TBI	Not TBI	TBI	Not TBI	TBI
1	155	163	184	178	180	174
2	157	160	163	188	185	170
3	160	164	190	174	172	183
4	158	168	177	191	186	189
5	165	158	170	186	161	176
6	159	160	181	188	185	189

The weight gain of the rats after 8 days of treatment indicated that the rats were healthy.

**Figure 2.**
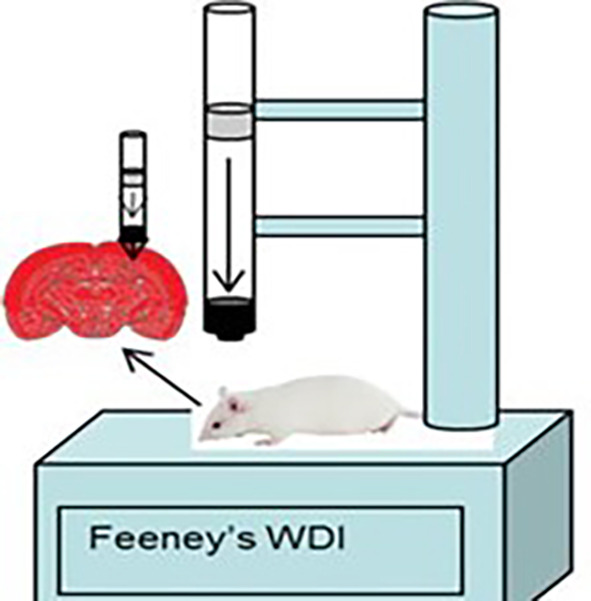
Feeney weight drop mode. ^9^

**Figure 3.**
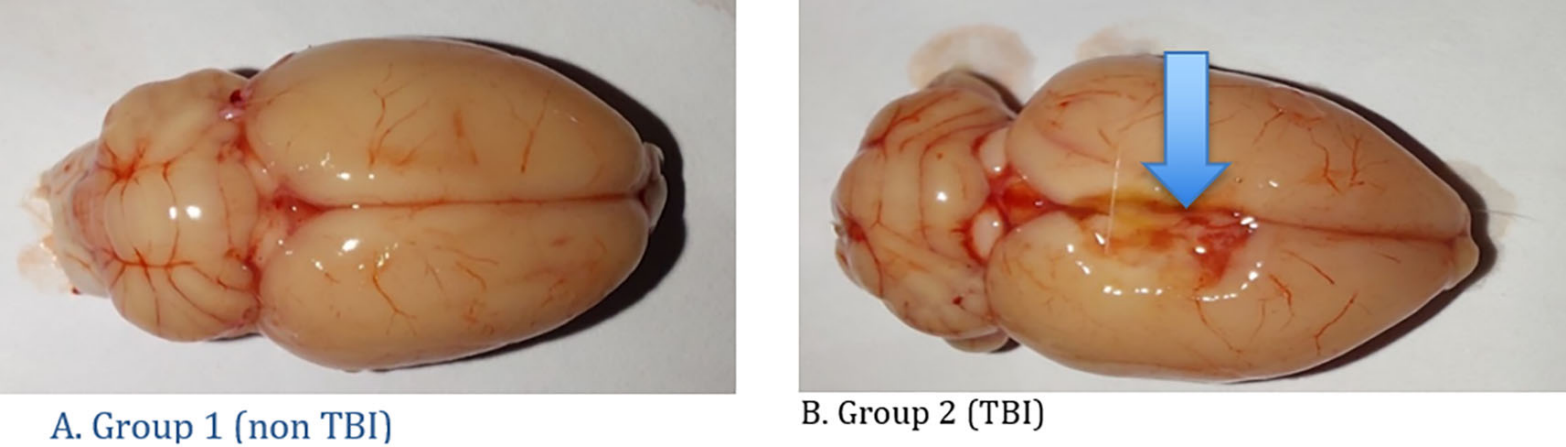
Macroscopic findings of the rat brain. A. Group 1 (non TBI): It was seen that there were no changes in the rat brain and there were no lesions in the brain; B. Group 2 (TBI): There is a lesion in the right side of the brain due to head trauma (blue arrow).

**Figure 4.**
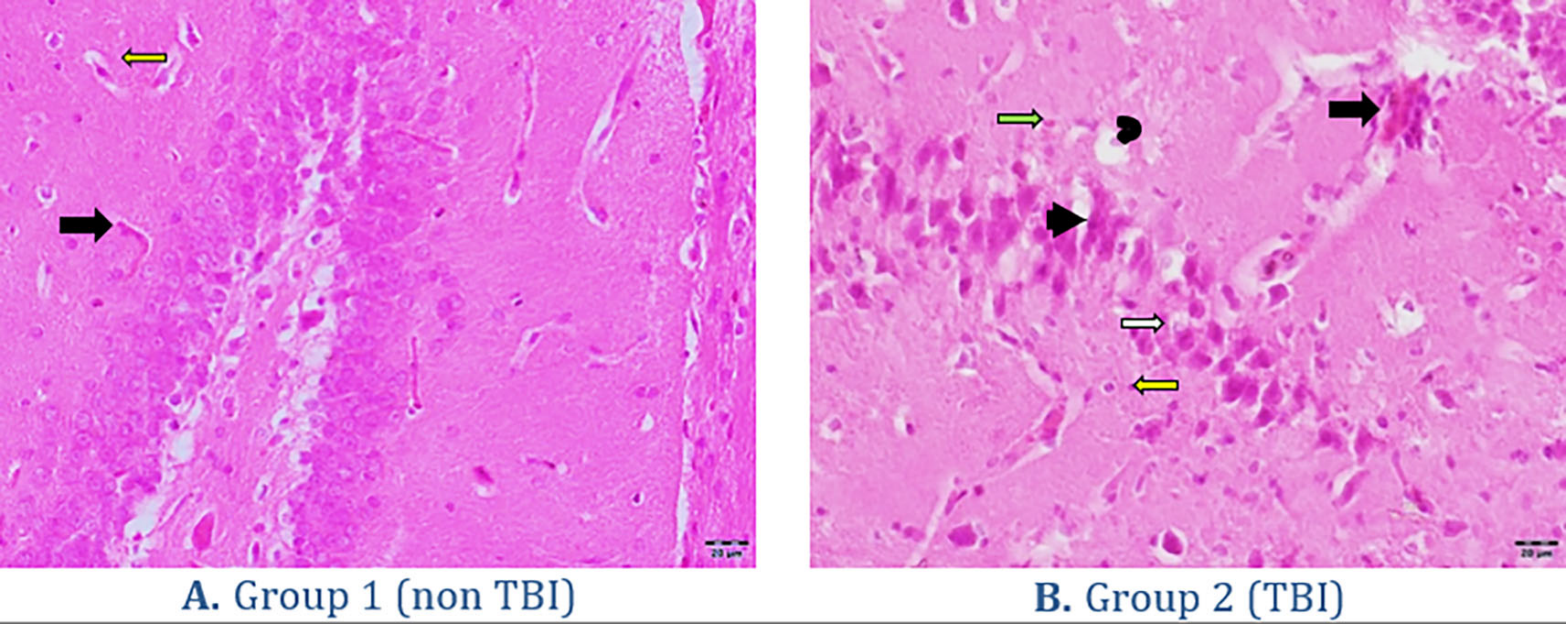
Histopathology of brain organs. (A) represents the group without treatment. (B) represents the treatment group with traumatic brain injury. Black arrow = congestion, white arrow = degeneration, headed arrow = necrosis, concave arrow = apoptosis, yellow arrow = hemorrhage and green arrow = inflammatory cells. Hematoxylin Eosin Staining with 400× Magnification.

**Table 2.** Quantities of degenerative processes, necrosis, congestion apoptosis, inflammatory cells and hemorrhage in the two groups.

Group	Rats	Histopathology changes per field of view (FV)					
		Degenerative	Necrosis	Apoptosis	Congestion	Inflammatory cells	Hemorrhage
Not TBI	1	20.5	5	3.5	0.5	6	3
	2	17.5	7	3.5	0.5	5	1.5
	3	21	5.5	2.5	1	3	3
	4	25.5	5	1.5	1.5	4.5	2
	5	21.5	7	3.5	1.5	5.5	4
	6	21	5	3	0.5	3.5	1
TBI	1	48.5	33	18	3	12.5	8.5
	2	46.5	41	19.5	2	9	11.5
	3	51	39	17.5	2.5	12.5	11
	4	49.5	35	19	2.5	12	13
	5	52	36.5	17	2.5	13	9.5
	6	43	35.5	19	2.5	9.5	7

**Table 3.** Average (±standard deviation) results of observations of histopathology changes in the brain organs in each treatment.

Group	Findings	Mean	SD	Median	Min.	Max.	p *	P **	P ***
Not TBI	Degenerative	21.17	2.56	21.00	17.50	25.50	0.352	<0.001	
TBI		48.42	3.28	49.00	43.00	52.00	0.730		
Not TBI	Necrosis	5.75	0.99	5.25	5.00	7.00	0.014		<0.001
TBI		36.67	2.89	36.00	33.00	41.00	0.860		
Not TBI	Apoptosis	2.92	0.80	3.25	1.50	3.50	0.070	<0.001	
TBI		18.33	0.98	18.50	17.00	19.50	0.557		
Not TBI	Congestion	0.92	0.49	0.75	0.50	1.50	0.035		0.001
TBI		2.50	0.32	2.50	2.00	3.00	0.101		
Not TBI	Inflammation	4.58	1.16	4.75	3.00	6.00	0.801		<0.001
TBI		11.42	1.72	12.25	9.00	13.00	0.077		
Not TBI	Hemorrhage	2.42	1.11	2.50	1.00	4.00	0.801	<0.001	
TBI		10.08	2.18	10.25	7.00	13.00	0.973		

* Shapiro-Wilk.

** Anova.

*** Kruskal Wallis Test.

Microscopically, there were differences between the two treatment groups as can be seen in Figure 3.

Histopathology findings in both groups of rats can be seen in Figure 4.

The histopathology changes in the brain organ in the non TBI group showed normal neuron cells ( Figure 4A). Changes in the TBI group showed a lot of damage to neuron cells that were necrotic and some experienced apoptosis, in addition to blood congestion (congestion), hemorrhage and visible degeneration of the brain ( Figure 4B). Quantitatively the process of degeneration, necrosis, apoptosis, congestion, inflammatory cells and hemorrhage in the five groups can be seen in Table 2.

The results of the statistical tests on histopathology changes in the form of degeneration, necrosis, congestion apoptosis, inflammatory cells and hemorrhage in all groups can be seen in Table 3.

The description of histopathology changes in the brain organs in the group of rat without TBI found that neuron cells looked normal although there were also degeneration (21.16 ± 2.56/FV), necrosis (5.75 ± 0.98/FV), apoptosis (2.91 ± 0.80/FV), congestion (0.91 ± 0.49/FV), inflammatory cells (4.58 ± 1.15/FV) and hemorrhage (2.41 ± 1.11/FV). Changes in the rat traumatic brain injury group showed a lot of damage to neuron cells in the form of degeneration (48.41 ± 3.27/FV), necrosis (36.66 ± 2.89/FV), apoptosis (18.91 ± 1.24/FV), congestion (2.50 ± 0.31/FV), inflammatory cells (11.41 ± 1.71/FV) and hemorrhage (10.08 ± 2.17/FV). Based on the results of statistical analysis, it can be seen that in all parameters there is a significant difference (p ≤ 0.001).

## Discussion

Traumatic brain injury is a condition of injury to the head caused by external mechanical forces which results in abnormalities in brain tissue. According to the path-mechanism of head injury, it is divided into primary injuries which are head injuries as a direct result of a trauma, which can be in the form of a direct impact or an acceleration-deceleration process of head movement or it can also be caused by a coup and counter-coup event. Secondary injuries are injuries that occur as a result of various pathological processes that arise as an advanced stage of primary brain damage in the form of bleeding, brain edema, ongoing neuronal damage, ischemia. and neuron-chemical changes. Abnormalities in brain tissue in humans can result in changes in physiological function of the brain and can lead to pathological and histopathology conditions of the brain. ^9^^–^^11^

Clinically, these changes in brain function can cause clinical symptoms in the form of periods of loss or decreased level of consciousness, signs of memory loss immediately before or after the incident, neurological deficits in the form of muscle weakness, balance disturbances, changes in vision, paralysis, sensory loss, aphasia, and so on, and there is a change in mental status at the time of injury such as confusion, disorientation, slow thinking, and so on. This disorder can be permanent or temporary. ^9^^–^^11^

In this study of the treatment of head injuries using the Feeney model, rats were dropped from a height of one meter with the right side as the fulcrum so that the right side of the brain experienced TBI. The desired sample is 12 rats. In this study, macroscopic rat brains showed the location of the lesion on the right brain. In TBI rats, bruises and abrasions appear on the surface of the brain, swelling (cerebral edema) on the surface of the brain, extensive subdural and subarachnoid hemorrhages, visible dilation of blood vessels, and visible bleeding from the cerebrum, cerebellum to the brainstem. ^12^

In rats that experienced TBI, bruises and abrasions appeared. Bruises indicate the location of a focal injury where the place is in contact with the surface of the blunt object that injured it. Blisters occur due to the displacement of the brain parenchyma and can also be combined with bruises on the surface of the brain. ^13^ In the TBI group rats appeared to have brain edema after 8 days of head injury treatment. Brain edema is an important risk factor for determining the prognosis of TBI, brain edema indicates an inflammatory reaction. ^12^ Bleeding and hematoma formation occur as a result of torn brain blood vessels during head injury. Bleeding into the subarachnoid space is common in cases of cerebral vascular trauma after head injury. Intraventricular and intracerebral bleeding are also common after trauma. ^12^^,^^13^

Microscopically, traumatic brain injury shows a lesion in the form of significant degeneration of neurons in the neocortical area with increased vacuolization. ^14^ The degenerating neuronal cells are characterized by bright eosinophilic cytoplasm with condensed nuclei, in addition, there is vacuolization inside the cells showing swelling/degeneration. Neuronal cells that experience death are characterized by shrinking cell nuclei and nuclei experiencing pyknotic or karyorrhexis. ^15^ Traumatic brain injury lesions in other cases also show extensive edema, neuronal necrosis, karyolysis and vacuolar changes, and widened inter-cell gaps. ^16^ In severe TBI, brain intraparenchymal hemorrhage may be found. Inflammation that occurs as a result of injury is a response from damaged tissue, triggering the release and activation of cytokines and chemokines as well as activation and proliferation of microglia. Elevated cytokines and other inflammatory mediators such as IL-1α, IL-6, and TNF-α were also confirmed in other studies of traumatic brain injury, in which the traumatic stress response was one of the causes. Metabolic disorders, including hypoxia and oxidative stress can lead to neuronal apoptosis, disruption of the blood brain barrier and brain edema following traumatic brain injury. ^17^^,^^18^

Cell degeneration is a type of cell damage that occurs in the mitochondria and cytoplasm due to a minor injury that can be repaired if the cause is removed. This cell degeneration is a decline in cell function that interferes with cell metabolism which is reversible. ^18^ In this study all treatment groups showed different numbers of degenerated cells, statistically the ANOVA test found significant differences (p < 0.001).

Cell necrosis is a permanent cell in the form of dead tissue. If the degeneration is reversible, then necrosis is irreversible, which means the dead cells cannot be repaired. With histopathology examination, the presence of dead cells can be determined so that if a healing process is needed immediately can be handled by removing the dead cells. In TBI there is an obstacle to the supply of blood and oxygen to the cells so that the cells die. This condition is caused by various things, such as injury, infection, extreme environment, to certain medical conditions that cause damage to the cells so that the cells die and can no longer function normally. ^18^^,^^19^ The statistical test results using the Kruskal Wallis test obtained a significant value (p < 0.001) as in Table 3. This p value indicates that there were differences from all TBI treatment groups in causing necrosis, statistically significant differences were found because the p value < 0.05.

Apoptosis is a component of the series of events following cerebral trauma and one of the main pathways leading to cell death. If necrosis occurs for some reason, apoptosis generally lasts a lifetime. ^20^ In this study all TBI treatment groups showed different numbers of cells undergoing apoptosis, statistically the ANOVA test found a significant difference (p = 0.001).

Congestion in general means there is blood damming in the tissue with a marked abundance of blood in the blood vessels in a particular region. Congestion is often also called hyperemia, when viewed microscopically the capillaries in the hyperemic tissue appear dilated and filled with blood. Cell congestion means there is congestion at the cellular level. ^19^ Investigating micro vascular congestion, blood vessels are carefully examined to see if they are small, medium or large containing red blood cells. ^21^ In this study on TBI rat, there were several variations in the level of cell congestion with significant differences for all groups. Statistical test results using the Kruskal Wallis test obtained a significant value (p = 0.001) as in Table 3. This p value indicates that there were differences from all TBI treatment groups in causing congestion, statistically significant differences were found because the p value < 0.05.

Inflammation is a natural response of the immune system when an injury or disease occurs. When rats experience TBI, they secrete inflammatory cells as a defense mechanism of the body that plays a role in the healing process of wounds which on histopathology examination are often called inflammatory cells. When mice get an infection due to TBI, there are many cells of the immune system that are involved, which then release various substances known as inflammatory mediators. ^22^ The statistical test results using the Kruskal Wallis test obtained a significant value (p = 0.001) as in Table 3. This p value indicates that there were differences from all TBI treatment groups in causing inflammatory cells to occur, statistically significant differences were found because the p value < 0.05.

Hemorrhage is the body’s response when injured by bleeding as a result of leaking blood vessels. ^23^ In this study all treatment groups showed different numbers of hemorrhagic cells, statistically the ANOVA test found significant differences (p < 0.001). In this study, quantitative differences were seen in degeneration, necrosis, apoptosis, congestion, inflammatory cells and hemorrhage in rat brain cells in each treatment. This shows a different inflammatory response from each treatment. The greater the number of degeneration, necrosis, congestion apoptosis, inflammatory cells and hemorrhage in rat brain cells, the greater the severity of inflammation caused by traumatic brain injury in rats.

Determining whether or not TBI is very important in the practice of forensic medicine. The results of histopathology analysis can be an alternative to determining causality in a short period of time at low cost. However, in the not TBI group, there were also histopathology changes indicating a different mechanism in the not TBI group. This study showed an increase in the level of cell damage in the TBI group compared to the not TBI group. Sequentially the comparison of the TBI group with the not TBI group is degeneration (2.28;1), necrosis (6.38:1), apoptosis (6.28:1), congestion (2.72:1), inflammatory cells (2.49:1) and hemorrhage (4.17:1).

The limitation of the study is that it has limited literature on premortem histopathology so that the discussion in this study is less than optimal. So further research is needed to increase the amount of literature.

This research is in line with studies conducted by Ibrahim et al., which stated that there were signs of inflammation in the form of haemorrhage and inflammation as well as necrosis in tissue as the body's response to significant antemortem stress, this also showed enzymatic changes in tissue. Meanwhile, for postmortem cells, there is tissue necrosis and severe cell damage. A combination of antemortem and postmortem histopathology showed changes in fatality in mice subjected to hypothermia. ^24^

This research is also in line with the study conducted by Al Salh & Al-Jameel, which stated the difference between antemortem and postmortem wounds, with antemortem characteristics being bleeding, increased macrophages and neutrophils, while postmortem showed tissue degeneration, bleeding and autolysis. ^4^

## Conclusions

We conclude that the damage to neuronal cells is more severe in premortem rats with traumatic brain injury, especially necrosis, apoptosis and hemorrhage. There is a significant difference between premortem histopathology findings in rats with and without traumatic brain injury which can be used for the benefit of forensic medicine in determining whether TBI is present or not. It can be said that to determine the presence of TBI, it is necessary to look more closely at the histopathology changes in the form of necrosis, apoptosis and hemorrhage. The results of this study are relevant in animal models, further research will be carried out to find their relevance in human subjects so that they are useful for forensic medicine. In relation to determining the mechanism of death due to traumatic brain injury, a histopathological examination is needed to ensure a stronger causal relationship.

## Data Availability

Zenodo. Master Data for Histopathology, https://doi.org/10.5281/zenodo.8260204. ^25^ This project contains the following underlying data:
•
MASTER DATA.docx. (Master Data for Histopathology) **Zenodo: ARRIVE** checklist for ‘Comparison between premortem histopathology findings in rats with and without traumatic brain injury: prospective application in forensic medicine’, https://doi.org/10.5281/zenodo.8351638. ^26^ Data are available under the terms of the Creative Commons Attribution 4.0 International license (CC-BY 4.0).
