# Supplementary material for: Comparison between premortem histopathology findings in rats with and without traumatic brain injury: prospective application in forensic medicine
Source: F1000Res. 2024 May 22;12:1311. Originally published 2023 Oct 11. [Version 2] doi: 10.12688/f1000research.140718.2 (PMC11399755; doi:10.12688/f1000research.140718.2)
Supplement: Peer review history

# Reviewer report: Reviewer response for version 2

**DOI:** 10.5256/f1000research.166842.r281450

**Published:** 2024-09-13

**Authors:** Ankita Guleria

**Affiliations:** 1 Panjab University,, Chandigarh, India

The authors were recommended to add some literature and compare the findings with them. But hardly any change is seen in the revised manuscript.

Is the work clearly and accurately presented and does it cite the current literature?

Partly

If applicable, is the statistical analysis and its interpretation appropriate?

Yes

Are all the source data underlying the results available to ensure full reproducibility?

Partly

Is the study design appropriate and is the work technically sound?

Partly

Are the conclusions drawn adequately supported by the results?

No

Are sufficient details of methods and analysis provided to allow replication by others?

Yes

Reviewer Expertise:

Forensic Science, Forensic Anthropology, Osteology,  Physical Anthropology.

I confirm that I have read this submission and believe that I have an appropriate level of expertise to confirm that it is of an acceptable scientific standard.

# Reviewer report: Reviewer response for version 2

**DOI:** 10.5256/f1000research.166842.r281451

**Published:** 2024-09-09

**Authors:** Putri Dianita Ika Meilia

**Affiliations:** 1 Persahabatan General Hospital, Jakarta, Indonesia

Thank you for the revisions.

Is the work clearly and accurately presented and does it cite the current literature?

Yes

If applicable, is the statistical analysis and its interpretation appropriate?

Partly

Are all the source data underlying the results available to ensure full reproducibility?

Yes

Is the study design appropriate and is the work technically sound?

Partly

Are the conclusions drawn adequately supported by the results?

Yes

Are sufficient details of methods and analysis provided to allow replication by others?

Yes

Reviewer Expertise:

Forensic medicine, forensic pathology, clinical forensic medicine, forensic epidemiology

I confirm that I have read this submission and believe that I have an appropriate level of expertise to confirm that it is of an acceptable scientific standard.

# Reviewer report: Reviewer response for version 1

**DOI:** 10.5256/f1000research.154105.r268142

**Published:** 2024-05-16

**Authors:** Ankita Guleria

**Affiliations:** 1 Panjab University,, Chandigarh, India

I would like to extend my sincere thanks to you for selecting me as a reviewer for F1000. I am truly honored to have this opportunity to contribute to the peer-review process. This is a very interesting topic with its importance in forensic medicine. I have read the manuscript and I have some suggestions for this manuscript. This study will sound technically more correct if following suggestions will be considered which I have found missing in the manuscript.

 I suggest authors to incorporate the following recommendations in their study:

1.  Authors should focus more on their introductions. This part needs to be strengthened and should provide a concise justification of the objective, goal, and significance of your study
2. *Rattus novergicus* is not italicized in the section under "Animal model." Authors should confirm the rat species whether it is ‘novergicus’ or ‘norvegicus’.
3.  Technically and scientifically, the discussion part is incorrect. Writers have only exclusively defined scientific terminology. I advise the authors to focus more on their discussion, specific research findings, and comparisons with other studies.
4. The conclusion has to be revised to reflect the forensic significance of this work and to be more specific. 

Is the work clearly and accurately presented and does it cite the current literature?

Partly

If applicable, is the statistical analysis and its interpretation appropriate?

Yes

Are all the source data underlying the results available to ensure full reproducibility?

Partly

Is the study design appropriate and is the work technically sound?

Partly

Are the conclusions drawn adequately supported by the results?

No

Are sufficient details of methods and analysis provided to allow replication by others?

Yes

Reviewer Expertise:

Forensic Science, Forensic Anthropology, Osteology,  Physical Anthropology.

I confirm that I have read this submission and believe that I have an appropriate level of expertise to confirm that it is of an acceptable scientific standard, however I have significant reservations, as outlined above.

# Response

**Published:** 2024-05-20

**Authors:** Taufik Suryadi

**Affiliations:** Universitas Syiah Kuala, Indonesia

Dear Prof.Ankita Guleria

 Thank you very much for your generous comment. I was greatly helped by your suggestions, and I revised my article manuscript according to your suggestions.

 1. At the end of the introduction section, I added some information:

 The aim of this study was to compare the results of premortem histopathological findings in animal model with or without traumatic brain injury. The target of the research is for forensic pathologists to be better able to provide evidence of traumatic brain injury to investigators so that the investigation process is optimally successful. The significance of this research is that it provides additional information for forensic pathologists in determining the mechanism of death if this research can later be applied to humans.

 2. Yes, we agree that the words "Rattus norvegicus" are not written in italics, I corrected them in our manuscript. We also stated that the correct one is "Rattus norvegicus" not "Rattus novergicus" and we standardized it in the article.

 3. At the end of the discussion section, I added some information from other research that is relevant to this research:

 This research is in line with studies conducted by Ibrahim et al., which stated that there were signs of inflammation in the form of haemorrhage and inflammation as well as necrosis in tissue as the body's response to significant antemortem stress, this also showed enzymatic changes in tissue. Meanwhile, for postmortem cells, there is tissue necrosis and severe cell damage. A combination of antemortem and postmortem histopathology showed changes in fatality in mice subjected to hypothermia.

 This research is also in line with the study conducted by Al Salh & Al-Jameel, which stated the difference between antemortem and postmortem wounds, with antemortem characteristics being bleeding, increased macrophages and neutrophils, while postmortem showed tissue degeneration, bleeding and autolysis.

 4. In the conclusion, I add information about the specifics of the findings:

 In relation to determining the mechanism of death due to traumatic brain injury, a histopathological examination is needed to ensure a stronger causal relationship.

 Best regards, 

 Taufik Suryadi

# Reviewer report: Reviewer response for version 1

**DOI:** 10.5256/f1000research.154105.r220553

**Published:** 2023-11-14

**Authors:** Putri Dianita Ika Meilia

**Affiliations:** 1 Persahabatan General Hospital, Jakarta, Indonesia

This is an interesting article detailing an experimental study about signs of traumatic brain injury in an animal model (rats). There are only some minor points that I would like to address:

1. To the best of my knowledge, this is not a case-control study. Rather, it is an experimental study using 2 independent groups. A case-control study is a form of observational study in which the starting point is 1 group of subjects who have a certain condition of interest and 1 group that does not, and then the exposure(s) or risk factors of interest are traced back.
2. The wording in the "statistical analysis part" is still in future tense, i.e., "If the data is not normally distributed, then the Kruskal-Wallis hypothesis test is carried out. If there is a difference, then the analysis is continued with a post hoc test to find out the different data pairs." Because at the time of writing, the authors should already have the data, that sentence should be changed into a more definitive form.
3. The conclusion as to whether the results of this study could benefit forensic medicine could be more clearly elaborated, e.g., what kinds of assumptions are needed to be able to draw an animal model-to-human inference.

Is the work clearly and accurately presented and does it cite the current literature?

Yes

If applicable, is the statistical analysis and its interpretation appropriate?

Partly

Are all the source data underlying the results available to ensure full reproducibility?

Yes

Is the study design appropriate and is the work technically sound?

Partly

Are the conclusions drawn adequately supported by the results?

Yes

Are sufficient details of methods and analysis provided to allow replication by others?

Yes

Reviewer Expertise:

Forensic medicine, forensic pathology, clinical forensic medicine, forensic epidemiology

I confirm that I have read this submission and believe that I have an appropriate level of expertise to confirm that it is of an acceptable scientific standard.

# Response

**Published:** 2024-05-20

**Authors:** Taufik Suryadi

**Affiliations:** Universitas Syiah Kuala, Indonesia

Dear Dr.Putri Ika Dianita Meilia

 Thank you very much for your generous comment. I was greatly helped by your suggestions, and I revised my article manuscript according to your suggestions.

1. Yes, I agree with your suggestion, that this research is experimental with two independent groups, I will revise it in the study design section.
2. Yes, I agree with you, I will revise the sentence on statistical analysis with: The research results were analyzed using the Shapiro-Wilk normality test. Statistical analysis was carried out using ANOVA (analysis of variance) on normally distributed data. For data that is not normally distributed, the Kruskal Wallis hypothesis test is carried out. Post hoc test analysis was carried out on different data pairs.
3. In the conclusion, I add information about: The results of this study are relevant in animal models, further research will be carried out to find their relevance in human subjects so that they are useful for forensic medicine. In relation to determining the mechanism of death due to traumatic brain injury, a histopathological examination is needed to ensure a stronger causal relationship.

Best regards

 Taufik Suryadi
